# Fumonisin B Series Mycotoxins’ Dose Dependent Effects on the Porcine Hepatic and Pulmonary Phospholipidome

**DOI:** 10.3390/toxins14110803

**Published:** 2022-11-18

**Authors:** Omeralfaroug Ali, Miklós Mézes, Krisztián Balogh, Melinda Kovács, Janka Turbók, András Szabó

**Affiliations:** 1 Agribiotechnology and Precision Breeding for Food Security National Laboratory, Department of Physiology and Animal Health, Institute of Physiology and Nutrition, Hungarian University of Agriculture and Life Sciences, 7400 Kaposvár, Hungary; 2 Department of Feed Toxicology, Institute of Physiology and Nutrition, Hungarian University of Agriculture and Life Sciences, 2100 Gödöllő, Hungary; 3 ELKH-MATE Mycotoxins in the Food Chain Research Group, Department of Physiology and Animal Health, Institute of Physiology and Nutrition, Hungarian University of Agriculture and Life Sciences, 7400 Kaposvár, Hungary

**Keywords:** fumonisins, mycotoxin, lipid, swine, phospholipid, sphingomyelin, fatty acid, lipid peroxidation, liver, lung

## Abstract

Male weaned piglets n = 6/group were fed Fumonisin B_1+2+3_ (FBs) mycotoxins at 0, 15, or 30 mg/kg diet for 3 weeks to assess the fatty acid (FA) composition of membrane lipid classes, lipid peroxidation, and histomorphological changes in the liver and lung. Growth performance and lipid peroxidation were unaltered, but histomorphological lesion scores increased in the liver. Linear dose–response was detected in liver phosphatidylcholines for C16:1n7, C18:1n9, and total monounsaturation and in lungs for C22:6n3, total n-3 and n-3:n-6, in pulmonary phosphatidylserines C20:0 and C24:0. Alterations associated with the highest FBs dose were detected in sphingomyelins (liver: total saturation ↓, total monounsaturation ↑), phosphatidylcholines (liver: total n-6 ↓, n-6:n-3 ↑; in lungs: total monounsaturation ↑, total polyunsaturation ↑), phosphatidylethanolamines (liver: total n-3 ↓; in lungs: total monounsaturation ↑ and n-6:n-3 ↑), phosphatidylserines (liver: n-6:n-3 ↑; in lungs: total saturation ↓, total polyunsatuartion ↑, and total n-6 and its ratio to n-3 ↑), and phosphatidylinositol (n-6:n-3 ↑; lungs: C22:1n9 ↑, C22:6n3 ↓, total saturation ↓, total monounsaturaion ↑). In conclusion, FBs exposures neither impaired growth nor induced substantial lipid peroxidation, but hepatotoxicity was proven with histopathological alterations at the applied exposure period and doses. FA results imply an enzymatic disturbance in FA metabolism, agreeing with earlier findings in rats.

## 1. Introduction

Mycotoxins are the secondary products that are generated from fungal metabolism. As of today, more than 400 metabolites have been identified; however, those that pose the greatest public health concerns have received the most attention. In a recent worldwide survey on mycotoxin presence in cereals, 100% of analyzed samples had 10 or more mycotoxins, of which 98% were infected by *Fusarium* genera [1,2]. Fumonisins, identified in 1988 [3], are mycotoxins that are mainly produced by *Fusarium* spp., namely *F. verticilloides* and *F. proliferatum.* Among the identified four groups (A, B, C, and P) of fumonisins, the B group (FBs: FB_1_, FB_2_, FB_3_, FB_4_, and FB_5_^c^) is the most studied due to its prevalence and health implications [4]. FBs have been detected in numerous cereal crops, although maize is regarded to be the most prevalent type for fumonisins [5]. Hence, FBs may pose a worldwide hazard to humans and animals through accessing their food and feed chains.

The adverse effects of FBs on animals have been documented in numerous species, including horses, rats, mice, pigs, rabbits, cattle, sheep, chickens, ducks, and primates [6]. Commonly, despite FBs’ low absorption rate [7], pigs are majorly exposed to FBs through the diet (composed of contaminated cereals, especially maize). Under FBs exposure, doses above and below EU established limits for pigs [8], histopathological abnormalities in the heart, lung, liver, kidney, and spleen have been recorded, whilst pulmonary edema (PPE) and cardiac dysfunction are the most frequent clinical signs in pigs [9]. Furthermore, FBs have been shown to disrupt the porcine intestinal barriers [10] and the membranes of erythrocytes by altering their lipid composition and Na^+^/K^+^ ATPase activity [11].

The structure of fumonisins is the driving force underlying the aforementioned adverse consequences. Fumonisins are similar to sphinganine (Sa) and thus disrupt sphingolipid metabolism, being ceramide synthase (CerS) inhibitors. It has been reported that FBs alter the ratio between sphingoid bases in a dose-dependent manner, resulting in a high sphinganine/sphingosine ratio (Sa/So) that modifies cell signaling, such as growth and differentiation [12]. In urine and serum, alteration in the Sa/So ratio has been established as a reference biomarker for FBs exposure, as has the sphinganine-1-phosphate (Sa-1-P) level, which is also an efficient biomarker. Other molecular modes of action, including oxidative stress, activation of endoplasmic reticulum stress, modulation of autophagy, and the alteration of DNA methylation, can be involved in the FBs’ toxic effect on tissues and/or cell lines [13].

The literature indicates that FBs are harmful substances to the liver and lungs in pigs and mice [9,14]. However, the lungs and liver are the primary target organs for FBs in swine. FBs have been shown to trigger tumor necrosis factor-α expression in lungs of mice [14], to interfere with activities of specific CerS forms in porcine liver [15], and to quantitatively and qualitatively alter the porcine-hepatic and lung membrane lipids [15,16,17]. Thus, it is important to highlight that the available literature lacks data on the effects of FBs on the various bilayer-glycerophospholipid fractions and does not account for dose response influence. On the other side, few in vivo and in vitro (normal or carcinogenic lines) studies are available on FBs’ effects on the rat hepato-membrane lipids, including data on glycerophospholipids with a major focus on phosphatidylcholine (PC) and phosphatidylethanolamine (PE) fractions [18,19,20,21,22,23,24]. These studies on rat liver provided data on alterations in the microsomal polar lipids upon exposure to different high doses of FBs (below acute toxicity). FBs, particularly FB_1_, can make the membranes highly susceptible to oxidation and promote the free radical-initiated lipid peroxidation. The higher relative oxygen diffusion–concentration products, as well as the increase of membrane permeability, may induce oxidative stress and cell damage [25].

Hence, this in vivo study mainly aimed to assess whether weaned piglets are sensitive to the oral FBs sub-acute exposure by investigating the dose-dependent effect of FBs on the cell membrane phospholipidome (namely, the sphingomyelin (SM), PC, PE, phosphatidylserine (PS), and phosphatidylinositol (PI)) from the liver and lungs. Furthermore, our study provides data on the histomorphological structure and lipid peroxidation status of the liver and lungs of weaned piglets.

## 2. Results

### 2.1. Animal Growth Performance

None of the FBs doses (15 and 30 mg/kg diet) were able to induce a remarkable modification in the animals’ final body weight or gain, absolute and relative liver and lung weights, feed intake, or feed conversion efficiency (Table 1).

### 2.2. Sphingomyelin Fatty Acid Profile

Fatty acid profile of the **liver** sphingomyelin (SM) FAs is shown in Table 2. Among the detected saturated FAs, C12:0 (lauric acid), C20:0 (arachidic acid), and C22:0 (behenic acid) proportions responded to the 30 mg FBs/kg diet exposure, leading to a proportional depletion in arachidic acid and an increase in lauric and behenic acids, relative to the control. In the calculated unsaturation index (UI) and average chain length (ACL), we found no significant difference between the treatments.

In the **lung** sphingomyelins (Table 2), in comparison to control, the highest dose of FBs increased the proportions of C14:0 (myristic acid) and C22:1n9 (erucic acid). In contrast, the C24:0 (lignoceric acid) proportion decreased in piglets fed 30 mg FBs/kg diet. For the calculated indices, totals of unsaturation (UFA) and monounsaturation (MUFA) were decreased in animals fed the highest dose of FBs, as well as the UI.

**Figure 1:**
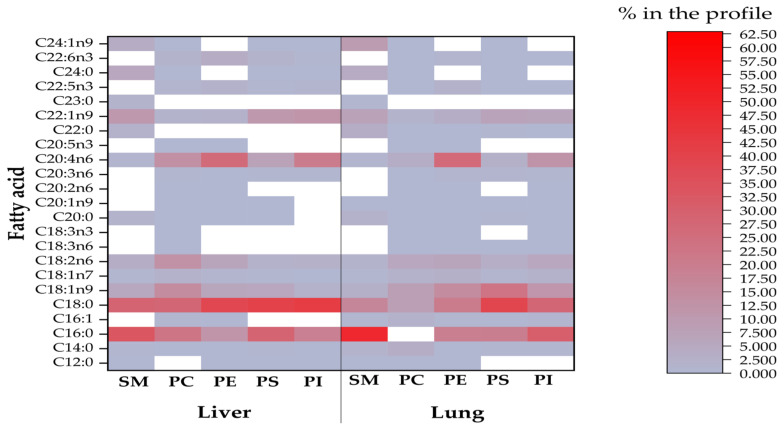
The compositional data of the FAs of all phospholipids from investigated liver and lungs (SM = sphingomyelin; PC = phosphatidylcholine; PE = phosphatidylethanolamine; PS = phosphatidylserine; PI= phosphatidylinositol). The FA proportion increases with color intensity, whereas the white color represents FA methyl esters below the detection limit of the GC.

**Figure 2:**
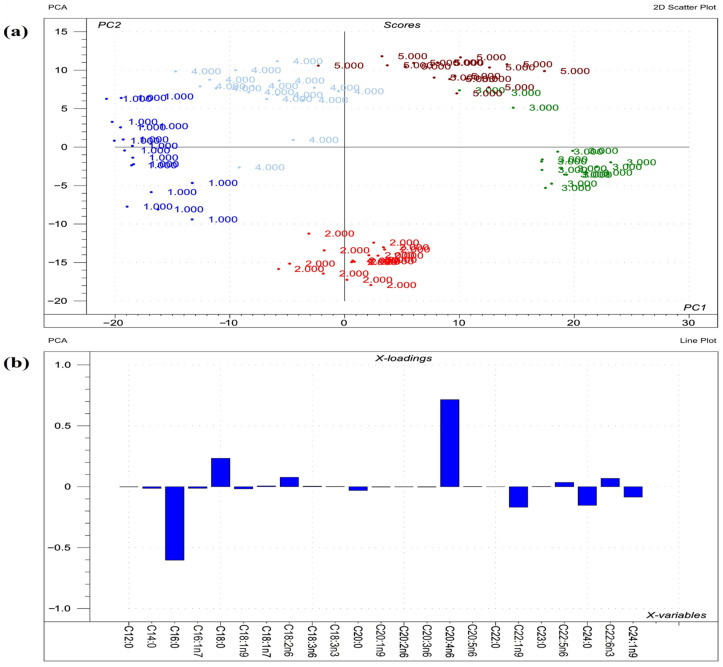
Results of PCA performed on the raw compositional data of the FAs of phospholipids from liver. (**a**) Score plot describes the orientation of the phospholipid classes from liver (1 = sphingomyelin; 2 = phosphatidylcholine; 3 = phosphatidylethanolamine; 4 = phosphatidylserine; 5 = phosphatidylinositol) in the plane of the first and second principal components (PC1 and PC2, respectively), where PC1 and PC2 are influenced by the multivariate data of FA of the organ phospholipids. PC1 and PC2 explain 57% and 28% of the total variance of FAs of the phospholipids, respectively. From the PCA, the polar FA pool of phospholipids from liver provided a perfect spatial separation of groups, referring to variation in their FA profiles. (**b**) Loading bar graph of the PC1 shows the contribution of the individual FAs of hepatic tissue to the newly developed latent variable; the higher the loading value, the greater is the impact of the respective FA’s variance on the variance of PC1. From the loadings, the remarkable FAs that contributed to variance between organs are C20:4n6, C16:0, C18:0, C22:1n9, and C24:0.

**Figure 3:**
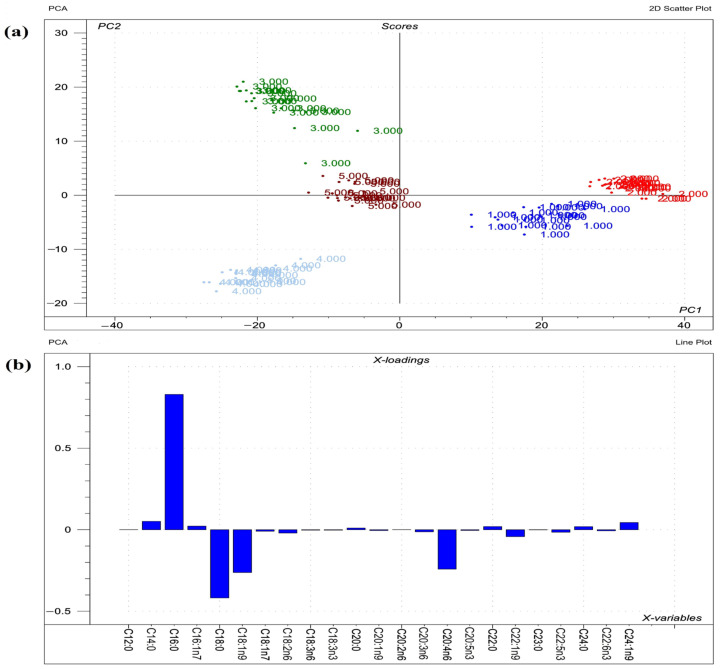
Results of PCA performed on the raw compositional data of the FAs of phospholipids from lungs. (**a**) Score plot depicts the orientation of the phospholipid classes from lung (1 = sphingomyelin; 2 = phosphatidylcholine; 3 = phosphatidylethanolamine; 4 = phosphatidylserine; 5 = phosphatidylinositol) in the plane of the first and second principal components (PC1 and PC2, respectively), where PC1 and PC2 are influenced by the multivariate data of FA of the organ phospholipids. PC1 and PC2 explain 73% and 18% of the total variance of FAs of the phospholipids, respectively. From the PCA, the polar FA pool of phospholipids from lung provided a perfect spatial separation of groups, referring to variation in their FA profiles; (**b**) Loading bar graph of the PC1 shows the contribution of the individual FAs of lung tissue to the newly developed latent variable: the higher the loading value, the greater is the impact of the respective FA’s variance on the PC1 variance. From the loadings, the remarkable FAs that contributed to variance between organs are C16:0, C18:0, C18:1n9, and C20:4n6.

**Figure 4:**
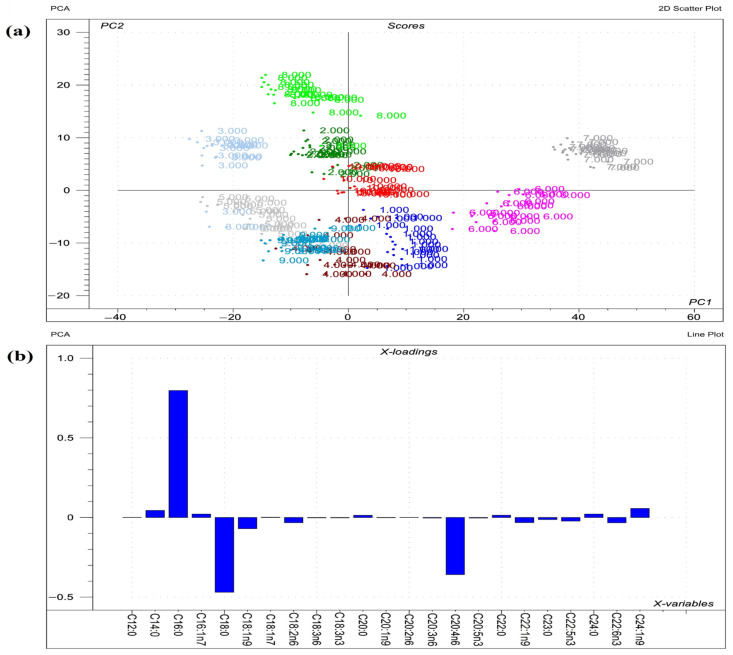
Results of the principal component analysis (PCA) performed on the raw compositional data of the fatty acids (FA)s) of phospholipids from organs. (**a**) Score plot depicts the orientation of the phospholipid classes from various organs (1 = liver sphingomyelin; 2 = liver phosphatidylcholine; 3 = liver phosphatidylethanolamine; 4 = liver phosphatidylserine; 5 = liver phosphatidylinositol; 6 = lung sphingomyelin; 7 = lung phosphatidylcholine; 8 = lung phosphatidylethanolamine; 9 = lung phosphatidylserine; 10 = lung phosphatidylinositol) in the plane of the first and second principal components (PC1 and PC2, respectively), where PC1 and PC2 are influenced by the multivariate data of FA of the organ phospholipids. PC1 and PC2 explain 64% and 17% of the total variance of the membrane FAs of the phospholipids, respectively. From the PCA, the polar FA pool of phospholipids from liver and lungs provided a perfect spatial separation of groups, referring to variation in their FA profiles; (**b**) Loading bar graph of the PC1 shows the contribution of the individual FAs from tissues to the newly developed latent variable; the higher the loading value, the greater is the impact of the respective FA’s variance on the variance of PC1. From the loadings, the remarkable FAs that contributed to the variance between organs are C16:0, C18:0, C20:4n6, C18:1n9, and C24:1n9.

Visualizations of the most pronounced FAs responsible for variations between phospholipid FA profiles are presented in Figure 1, Figure 2 and Figure 3, as well as Figure 4, which expresses the proportional presence of each FA in the various lipid fractions, based on color intensity.

### 2.3. Phosphatidylcholine Fatty Acid Profile

Table 3 presents the FA profiles of phosphatidylcholine (PC). In the **liver**, all animals subjected to FBs exhibited a proportional increase in myristic acid. At the highest FBs exposure, C18:0 (stearic acid), C18:2n6 (linoleic acid, LA), C20:4n6 (arachidonic acid, AA), total polyunsaturation (PUFA) and overall omega-6 (n-6) FAs proportions decreased. Contrastingly, within the same group, proportions of C18:1n9 (oleic acid), C16:1n7 (palmitoleic acid), and total MUFA levels increased. In piglets fed 15 mg FBs/kg diet, an elevated proportion of C18:3n3 (α-linolenic, ALA) was detected, but lower proportions were detected in C22:5n3 (docosapentaenoic acid, DPA) and total n-3 FAs. None of the other calculated indices were different from the control.

In the **lung** PCs, FBs’ exposures altered the FAs and their derived indices (Table 3). The proportions of C20:2n6 (eicosadienoic acid), DPA, C22:6n3 (docosahexaenoic acid, DHA), and the n-3 FAs total decreased in all intoxicated groups. In comparison with the control, proportions of lauric, myristic oleic and C22:1n9 (erucic acid) acids, total MUFA, and the ratio of n-6 to omega-3 FAs (n-6:n-3) were increased in lungs at 30 mg FBs/kg diet, but stearic acid and C18:1n7 (vaccenic acid) proportions decreased. Total saturation (SAT) decreased in the 15 mg FBs/kg group compared to the control and 30 mg FBs-setting, while the 15 mg/kg diet decreased proportions of C18:3n6 (γ-linolenic), C20:3n6 (dihomo-γ-linolenic acid), AA, total n-6 FAs, and UI, compared to control.

When linear dose-response was examined in the **liver PC** (Table 4), the marked increases of palmitoleic acid, oleic acid, and total MUFA were responsive with R^2^ > 0.6. Linear fittings with reliable R^2^ were attained for DHA, a total of n-3 FAs, and the ratio of n-6 to n-3 in the lung (Table 4). 

### 2.4. Phosphatidylethanolamine Fatty Acid Profile

The FA profile of the **liver** phosphatidylethanolamine (PE) fraction can be seen in Table 5. None of the treatments had a marked effect on the saturated FAs or the total SAT. FBs-fed animals showed a higher proportion of palmitoleic acid, relative to control. For oleic acid, only the highest FBs-setting increased its proportion, although the total MUFA concentration was not altered. The total n-3 FAs and ACL were decreased in piglets fed 30 mg FBs/kg diet as compared to control. 

In the **lung** PE (Table 5), both applied FBs doses increased the behenic acid proportion; nevertheless, the overall SAT was not altered. The 30 mg FBs/kg diet increased proportions of palmitoleic acid, LA, erucic acid, and total MUFA as compared to control. We found no significant difference in the other calculated indices (totals of SAT, UFA, n-3, and n-6, as well as UI and ACL). 

### 2.5. Phosphatidylserine Fatty Acid Profile

The phosphatidylserine (PS) FA composition results from the **liver** are shown in Table 6. Fatty acids where FBs-contaminated diets caused intergroup differences were lauric acid (increased), C24:1n9 (nervonic acid, increased), C20:1n9 (eicosenoic, decreased), and DPA (decreased). The n-6:n-3 ratio increased in the group with the highest FBs exposure, while none of the other presented calculated indices (total MUFA, total n-3 FAs, UI, and ACL) were altered. 

The FA profile of **lung** PS (Table 6) showed proportional depletions in stearic, arachidic, behenic and lignoceric acids, as well as in DHA and total SAT, a result of FBs’ exposures (15 and 30 mg/kg diet). In contrast, both FBs’ treatments displayed high proportions of LA, dihomo-γ-linolenic, and arachidonic acids, and consequently for the total n-6 FAs and total PUFA. The total MUFA was not altered, although the proportion of erucic acid increased in the 30 mg/kg diet group. 

As shown in Table 4, the FAs in the lungs with reliable linear fittings were behenic and lignoceric acids (R^2^ > 0.6). 

### 2.6. Phosphatidylinositol Fatty Acid Profile

For the liver phosphatidylinositol (PI) FA profile (Table 7), the comparison between control and FBs-treated groups revealed that the DPA, DHA, nervonic acid, and total n-3 FAs proportions decreased, and the n-6:n-3 ratio increased in all FBs-intoxicated piglets. The 15 mg FB/kg diet-associated differences were proven in lauric and stearic acids, and total SAT; the proportions were higher, while, within the same group, the proportions of erucic and total MUFA decreased.

In the lung-PI (Table 7), administration of 30 FBs/kg diet increased the proportions of erucic acid and total MUFA, compared to control. In contrast, proportions of stearic acid, DHA, and total SAT decreased in piglets fed on the highest dose of FBs. 

### 2.7. Antioxidants and Lipid Peroxidation

Table 8 shows the antioxidant and lipid peroxidation status of the liver and lungs. Regardless of the FBs dose applied and the investigated organ, no intergroup difference was detected in any of the investigated antioxidant parameters (reduced glutathione (GSH) content and glutathione peroxidase (GPx) activity) or in the end product of lipid peroxidation (thiobarbituric acid reactive substances (TBARS) expressed as malondialdehyde (MDA)).

### 2.8. Pathological Assessment 

**Figure 5:**
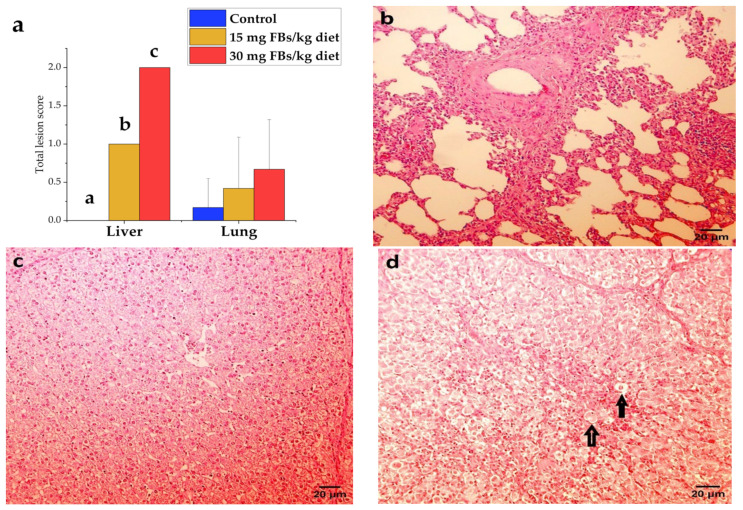
(**a**) Total lesion scores of liver and lungs recorded in experimental piglets (n = 6 animals/treatment, whereas columns represent means, and bars represent the standard deviation. The letters a, b, c above the bars indicate significant differences). (**b**) Lung of a healthy pig with mild lymphocytic and histiocytic infiltration in connective tissue (hematoxylin–eosin, 200 ×, scale bar = 20 µm). (**c**) A healthy piglet liver from control, where the cytoplasm of hepatocytes is finely granulated due to high glycogen content, resulting in an intense stain (hematoxylin–eosin, 200 ×, scale bar = 20 µm), although a PAS stain would be necessary to confirm our observation. (**d**) The liver of a highly FBs intoxicated piglet (30 mg/kg), where the glycogen content decreased in the hepatocytes’ cytoplasm and a high frequency of necrotic (rounded, faintly stained) hepatocytes (↑) detected (hematoxylin–eosin, 200 ×, scale bar = 20 µm).

During the study, mortality did not occur. Based on necropsy data, only one animal in the 30 mg FBs/kg group showed pale liver. In the lungs, slight vasodilatation and hyperaemia in the mesenterium were observed in some piglets from control and FBs-treated animals. The histological assessment, expressed as a total lesion score, of the liver and lungs is shown in Figure 5. In liver, the total-lesion score responded positively to the applied FBs level, expressing low cellular glycogen, hepatocyte necrosis, as well as swelling and proliferation of the mononuclear phagocyte system (MPS). Furthermore, the liver provided a dose-dependent response, in which 15 and 30 mg FBs/kg diet expressed mild and moderate intoxication, respectively. Despite the fact that lesions were found in the lungs (Figure 5a), no marked differences were found in the total lesion score among the groups (detected total scores were not above the mild toxicity level). The PPE was only recorded in a piglet fed on a 30 mg FBs/kg diet, in which the lung was expressed as heavy, swollen, pale, and doughy.

## 3. Discussion

### 3.1. Animal Performance

Animal growth performance is generally a basic but very complex physiological trait that is influenced by the most adverse stimuli. In this study, higher FBs’ doses (15 and 30 mg/kg diet) than the maximum proposed limit value for pigs (5 mg/kg diet) [26] were administered. 

Despite the fact that FB_1_ has been reported to impede pig growth [27] and induce anorexia [28,29,30], the highest FBs dose in this trial did not compromise growth (weights of the whole body and organs), feed intake, or feed conversion efficiency. Our insignificant finding of feed intake is not similar to those reported earlier [28,29,30]. We refer this inconsistency to the differences in experimental settings; applied dose, exposure period, genotype, age, and mycotoxin source form and purity. In regard to the growth perturbation, similar patterns to ours were reported in the studies of 12.2 mg FB_1_ + FB_2_/kg diet for 4 weeks [8]; 20 mg FB_1_/kg for 10 days [17]; 7.2, 14.7, 21.9, 32.7, and 35.1 FB_1_ + FB_2_ mg/kg diet for 4 weeks [31]; 25.1 mg FB_1_ + FB_2_ + FB_3_/kg diet for 42 days [32]; and 25.1 mg FB_1_ + FB_2_ + FB_3_/kg diet for 42 days [33]. Our maximum FBs-dose settings (cal. 26.17 mg FBs/kg^−1^ of final body weight during the trial) and period of exposure are lower than those of [33]; consequently, no remarkable variation in animal performance was predicted.

It is important to highlight that the lung weight (abs. or rel.) was not affected, as well as the PPE was only documented in a single intoxicated piglet. Therefore, the unaffected lung weight is a consequence of the absence of severe toxicity. There is a visible tendency in the toxin dose associated with lung weight (possibly indicating edema commencement) and body weight gain, but none was proven to be statistically significant. We keep the premise that prolonged exposure and a large group size (n > 6) will reveal minimal variances and lead to precise outcomes.

### 3.2. Sphingomyelins

The SM is the most abundant form of sphingolipids in cellular plasma membranes, accumulating primarily in the outer membrane leaflet. The biosynthesis of SMs is dependent on ceramide production, a key intermediate of sphingolipid metabolism and a major precursor of long-chain FA and complex sphingolipids. Relatively, the major components of its structure are rather long saturated chains (16:0, 18:0, and 24:0), although in mammalian spermatozoa, very long-chain (24:0 to 34:0) PUFA have been reported [34]. Our results are corroborated by the findings of [34], since we detected overall saturation levels exceeding 70 % in SMs from the liver and lungs.

Once ceramide is a precursor to SM synthesis, it is relevant to discuss the FBs’ effects on ceramide. In mammals, ceramide synthesis is catalyzed by the ceramide synthase enzymes (CerS; not determined in this study), a family consisting of six isoforms varying in acyl chain length interval specificity [35]. In the liver, 30 mg FBs/kg feed elevated the proportions of lauric (approx. two-fold) and behenic acids (1.5-fold), while decreasing the proportion of arachidic acid without altering overall SAT. This confirms the findings of [15] in piglet liver, when 1.5 mg FB_1_/kg body weight for 9 days increased the level of SM-d18:1/22:0. These patterns may be indicative of high ketoacyl-CoA synthase activity (markedly increased SM-C22:0/C20:0, data not shown) and/or inhibition of CerS activities in the liver due to FBs exposure. Remarkably, the upregulation of long and very long side-chained (such as C22:0) ceramide molecular species has been involved in apoptosis regulation, with CerS4 and CerS6 activities postulated to be implicated [36].

On a qualitative and quantitative scale, FBs’ effects on lung SM were more pronounced. Total SAT dropped concurrently with myristic acid (although the lignoceric acid proportion declined, presumably implicating lower CerS3 activity), whereas erucic acid and the overall MUFA level increased proportionally. Indeed, it is difficult to hypothesize precise changes in the activities of CerS isoforms since their affinity for FB_1_ is not uniform and the fraction and ratio across isoforms have not been determined. Similar findings have been reported for erucic acid [15]. It has been shown that erucic acid and MUFA in general have a protective effect against cytotoxicity, especially in cancer cell lines [19,37]. On the other hand, oleic acid has been linked with metabolic and inflammatory lung diseases [38]. Thus, we hypothesize that the large proportions of erucic and oleic acids in the lungs possibly resulted in diverse stimuli; a protective mechanism and an injury trigger, respectively. 

Modifications in the hydrocarbon chain length lead to variations in the bio-physicochemical properties of compounds, although behavioral changes are not fully comprehended. FBs are known to negatively affect the biophysical properties of cellular membranes [19]. In the case of high chain asymmetry, mismatch compensation can occur, and one such effect in the lipid bilayer is the establishment of chain interdigitation [39], which has been demonstrated in SM [40] and asymmetric ceramides [41]. We thus suppose that modifications in the SM fatty chain lengths were related to the interaction of lipids in the bilayer, most likely via chain interdigitation, in order to preserve or alter the physicochemical properties of the cell membrane.

### 3.3. Phosphatidylcholines

In the PC fraction, 30 mg FBs-exposure increased the myristic acid proportion (approx. two-fold in the liver and lungs). In contrast, stearic acid diminished in liver and lung, which was concomitant with the increase of oleic acid. These findings are consistent with reported findings in piglet liver-total PL (in vivo by FBs exposure at EU permitted value for 21 days [16]) and rat liver-PC fraction (in vivo by i.p. FB_1_ equal to 20, 50, and 100 mg/kg diet for days [18]; and 250 mg FB_1_/kg diet for a 21-day period [22], and in vitro by primary hepatocytes exposed to 25, 75, 150, 250, and 500 µM FB_1_ [21]). The low stearic acid proportions in the liver and lungs were most likely a consequence of the high activity of stearoyl co-enzyme A desaturase (SCD); a key enzyme mediating the desaturation of stearic acid [42]. We noticed high ratios of C18:0/C18:1n9 in the studied tissues, as well as C16:0/C16:1n7 in lung, indicating high SCD activities that likely exhibited antioxidant qualities against the FBs’ cytotoxicity. However, controversial patterns reported the adverse effects of MUFA in subsequent studies [43,44]. The endogenous pulmonary surfactant is predominantly formed from the reuptake of degraded PC, whereas 10–14% is synthesized via de novo routes [45]. Modifications in the lung-PC-FAs can have a genuine impact on the lung state. Oleic acid has been found to trigger pulmonary injury and inflammation through disrupting the Na^+^/K^+^-ATPase, Na^+^ channeling, membrane docking, and G-protein coupled receptor activities [38]. In our study, histological changes in the lung were minimal, suggestive of a potential role for elevated oleic acid and/or the low C18:0/C18:1n9 as early bio-indicators of the lung’s cytotoxicity.

Due to elevated proportions of palmitoleic and oleic acids in the liver (in a dose–response manner), and oleic and erucic acids in the lungs, the total MUFA levels increased in the liver and lungs of the 30 mg FBs/kg diet group. These findings support the results of [16] in piglets when the liver total PLs’ MUFA was increased, proposing a compensation mechanism for the depletion of total SAT that is responsible for membrane rigidity. In our case, the total MUFA markedly compromised both SAT and PUFA levels in the liver (altered SAT/MUFA and PUFA/MUFA, data not shown), and only PUFA in the lung (altered PUFA/MUFA). Rather than merely ensuring membrane rigidity maintenance, we assume MUFA elevation is likely to modify transmembrane signaling and the cell cycle. MUFA has been reported to suppress lipogenesis and enhance glucose sensitivity [46], but none of these were examined in this design. 

The highest dose of FBs decreased overall levels of n-6 and n-3 FAs in PCs of the liver and lung, resulting in low and high n-6:n-3 ratios in the organs, respectively. These findings were mainly consequences of the depletion of LA and AA in the liver PCs, as well as the lung-PC’s DPA and DHA in a dose response manner. Similar findings were reported [18,22] for AA, DPA, and DHA of PCs from different subcellular fractions of the rat liver membrane. Our liver-PC findings for AA, overall n-6 FAs, and its ratio to n-3 FAs seem to be inconsistent with those reported in piglets fed on a 20 mg FB_1_/kg diet for 10 days [17] in the total PL pool from porcine liver. In our work, separated PC was the investigated fraction, not membrane polar lipids as a whole. Furthermore, we applied a longer exposure period as compared to [17]. The AA depletion in liver-PC indicates higher activity of the enzyme phospholipase-2 (PLA_2_), most probably directing the substrate eicosanoids biosynthesis. PLA_2_ was shown to hydrolyse AA and DHA preferentially [47,48]. 

Meanwhile AA is a precursor in eicosanoid biosynthesis, DHA is involved in the regulation of prostanoid production. The proportionate declines in lung-PC’s DPA and DHA (ca. 3/4 proportional decrease) of the 30 mg FBs group largely indicated the progression of n-3 FA-derived prostanoid synthesis. The proportional decrease of DPA, DHA, and overall n-3 FAs by FBs has been reported in vitro and in vivo in the rat liver-PCs [20,22] and piglet liver-total PL [17], but has not yet been reported in the pig lungs. Thus, the novelty of this in vivo study is that we are reporting similar findings in the piglet lungs for the first time, in a dose-dependent manner, most likely due to the inhibition of rate-limiting delta-5- and delta-6-desatuarse enzymes (Δ5D and Δ6D) activities as reported in the rat liver [23]. Here, we are suggesting similar events in the porcine lung-PC fraction based on the marked findings in PC-C20:4n6/C20:3n6 (ΔD5, low in the highest FBs setting, data not shown), PC-C18:3n6/C18:2n6 (Δ6D, low in all FBs treated animals, data not shown), and PC-DPA and DHA (both depleted in lungs). 

### 3.4. Phosphatidylethanolamines 

PE, which accounts for 15–25% of mammalian cellular membrane lipids, is the second most abundant type of polar lipid in animal tissues. The efficacy of FBs in altering the rat liver-PE has been reported in numerous studies (in vivo and in vitro) on rats [18,21,22]. With respect to saturated FAs, unlike the finding in lung-PEs (FBs exposure decreased behenic acid), no change was detected in the liver-PE. In swine, effects of FBs on saturated FAs of the liver total PLs varied among studies; altered by FBs at the EU permitted limit for a 21-day period [16] and unaltered by 20 mg FB_1_/kg diet for 9 days [17]. Indeed, the doses and exposure periods applied in these studies differ. In comparison to rat liver models that reported alterations in PE-FAs, we mainly relate our non-observed effect to possible species-specific effects of FBs, lower FBs’ doses as compared to those of 250 mg FB_1_/kg diet for 14 days [21] and 250 mg FB_1_/kg diet for 21 days [22], and administration method and toxin purity as compared to applied intraperitoneally pure FB1 for 5 and 10 days [18]. 

In a similar manner to PC, proportions of monounsaturated FAs responded to FBs in a dose dependent manner, namely the palmitoleic (liver and lung (almost two-fold increase)), oleic (liver) and erucic (lung) acids. Similar findings have been reported in Chang cells [20]. Although monounsaturated FAs in liver-PE were responsive, their total sum was unaltered. Based on the available literature in swine, the MUFA findings on the total PL FA profile of porcine liver are controversial. A dose of 5 mg FBs/kg diet for 3 weeks increased MUFA level [16], whereas a 20 mg FB_1_/kg diet for 10 days did not alter its level [17]. These patterns are likewise reflected by the specific FBs-exposure period on the total MUFA, rather than the applied dose. On the other hand, in vivo studies on the rat liver-PE fraction have documented the accumulation of MUFA level induced by FBs exposure [21,22,23] through the up-regulation of the delta-9-desaturase (Δ9D) enzyme. Remarkably, we noticed these MUFA increments were attained at greater FBs’ doses (>100 mg/diet) than ours. Anyhow, our results revealed a high overall MUFA level in the lung, indicating the organ-specific response/sensitivity to FBs. Indeed, MUFA accumulation compromised the depletion of total PUFA, referring to modifications in cellular signaling and/or metabolism. The main compromised PUFA sub-group was the sum of n-3 FAs, which consequently increased the n-6:n-3 ratio, although no significant change was noticed in their individual FAs. 

A further marked modification was the decrease of total n-3 FAs and average chain length in the liver of piglets exposed to FBs, but these alterations were not dose-dependent, resulting in a markedly higher n-6:n-3 ratio. Similar patterns have been reported in piglet liver PL [17] and rat liver-PE fraction [21,22]. This class of lipid consists of crucial FAs that are involved in signaling or communication pathways within and between cells through altering membrane protein function and gene expression [49]. In general, the level of n-3 FAs in cellular membrane depends on their levels in the diet and their competition with n-6 FAs [50,51]. In our case, the alteration in total n-3 FAs of liver-PE was independent of oxidative stress (Table 8) and feed intake (Table 2), implying alterations in the enzyme activities involving remodulation of the membrane lipid fractions. Unlike the PCs, PE resides in the cytofacial leaflet; hence, the FA ratios between polar lipids in the membranes play a crucial role in cellular signaling, metabolism, proliferation, and death. We noticed that, under FBs exposure, the different tissues displayed distinct responses in terms of FAs indices among membrane lipids. The total n-3-PC/PE was found to increase in the liver, but it decreased in the lungs (data not shown). These findings indicate that the organ-specific response mostly depends on the cytotoxicity level and the ratio between lipid fractions. 

### 3.5. Phosphatidylserines

Ordinarily, PS mainly resides in the inner leaflet of the cellular membrane and is a minor class of total PLs (constituting 2–15% of the total membrane–lipid pool). To our knowledge, only one study has investigated the impact of FBs on the PS-FA profile of rat liver, but no data is yet available on PS from lungs of pigs exposed to FBs.

The liver dataset revealed no discernible FBs-effect on either total SAT or total MUFA of PS fraction. Similar patterns were reported [23] for SAT of liver-PS; however, FB_1_-doses of 50 and 100 mg/kg diet elevated the oleic acid proportion and total MUFA. In our hepatic and pulmonary results, total MUFA levels were not responsive. However, eicosenoic acid showed a marked proportional decrease along with the accumulation of nervonic acid (more than three-fold) in hepatocellular-PS, whereas in lung-PS, oleic acid decreased and erucic acid increased (1.5-fold more). Nervonic acid is produced from the carbon chain elongation of oleic acid by the cyclic addition of two carbon units provided by malonyl-CoA to the acyl chain [27]. Although nervonic acid is closely linked with the maintenance of nerve cells, it has also been observed to increase in patients with Alzheimer’s disease, psychosis, depression disorder, and cardiovascular disease [52], warranting further investigations into its related diseases and mode of action. In the lungs, we assumed that the accumulated erucic acid in PS could protect against pulmonary infections (see Section 3.8). Erucic acid has recently been shown to protect rats’ lungs from damage induced by the influenza A virus [53]. 

When we investigated the lung-PS FA profile, results indicated dose-dependent alterations in behenic and lignoceric acids (half proportional depletion). Stearic, arachidic, behenic, and lignoceric acids were the most responsive saturated FAs (all decreased), paralleling the low total SAT in intoxicated animals. Markedly, PUFA compromised the depletion of SAT (high PUFA/SAT), whereas contrastingly, no marked variation was detected in the SAT/MUFA. The depletion of SAT indirectly indicates that the FBs interfere with one or more of the four reactions of the elongation process in the endoplasmic reticulum and/or the insertion of an SAT type FA into position *sn-1* and *sn-2* of PS. The PS is a modulator of the membrane charge locality; as such, it is vital for neural transport and influences proteins involved in numerous metabolic processes (e.g., enzyme activation and apoptosis). PUFA accumulation in lung-PS was a result of the proportional increases in LA, dihomo-γ-linolenic, and arachidonic acids (n-6 FAs). Interestingly, the LA proportional increase by FB_1_ exposure has been reported in rat liver-PS [23]. The n-6 FAs play vital roles in numerous cellular molecular pathways, such as inflammation, signal transduction, and cellular proliferation and apoptosis. Thus, we assume that alterations in the lung-PS have a profound impact on PS-related signaling events in the lung. 

Our liver findings are relatively similar to those of [23], in which the n-6:n-3 FA decreased by FBs exposure, including extra the decrease of DPA proportion. Notably, similar patterns were noticed in lungs of intoxicated animals, associated with the proportional depletion of DHA. These patterns are independent of the lipid peroxidation process and dietary DHA intake (see Table 1 and Table 8), speculating that alternative molecular events are responsible for these observations. The level of PS in bilayers has been found to be strongly influenced by its n-3 proportion within the membranes. PS accumulation is very profound in neural tissue with abundant DHA concentration [54]. In this design, the PC, PS, and PI fractions all showed a proportionate depletion in DHA, underpinning a possible depletion from the cellular-PS proportion. Anyhow, our data is neither quantitative nor lyso-PS has been analyzed; thus, further studies are essential to support that proposal. 

### 3.6. Phosphatidylinositols 

The PI represents 2–12% of total polar-lipids in mammalian cells and subcellular membranes. Analyzing the PI-FA profile, apart from a few proportional changes, it is clear that linear dose-dependent trends are absent, most likely a result of the applied doses and exposure period. Based on the literature, two studies investigated the impacts of FB_1_ on the fatty profile of rat liver-PI [18,23]. 

Similar to the earlier report in rat liver [55], our piglet liver data revealed that stearic and arachidonic acids (together ca. 60% of the total PI-FAs pool) were the most abundant FAs in PI fraction. However, the lungs exhibited distinct patterns, with palmitic, stearic, arachidonic, and oleic acids being the most abundant FAs. Our dataset revealed that the proportions of PI-AA in the liver or lung remained unaffected, while the stearic acid proportion in the lung-PI decreased, depleting the total SAT level. Our AA finding is consistent with earlier results in the hepatic PI fraction of rats [18,23], but FBs’ effects on PI stearic acid are not yet clear. When a 250 mg FB_1_/kg diet for 21 days failed to alter the PI-stearic acid proportion [23], 10 days of pure FB_1_-intraperitoneal exposure (eq. 100 mg FB_1_/kg diet) decreased its concentration, as well as the total SAT level [18]. We speculate that the depletion in lung-PI-stearic acid proportion was related to elevated activity of Δ9D and elongation enzymes (not analytically determined). Indeed, we noticed no change in the lungs’ PI-stearic/oleic, but PI-stearic/behenic and PI-oleic/erucic were markedly lowered (data not shown). Notably, trials by [18,23] found high oleic acid and total MUFA concentrations, mostly attributed to the relatively high FBs’ dose compared to ours.

When comparing PS and PI from lungs of highly FBs-intoxicated animals, strikingly similar FA patterns were noticed; the erucic acid and overall MUFA compensated the decrease in total SAT. The fact that erucic acid was the major contributor to high MUFA indicates its possible essential role in protecting against lung injury. Recently, it was reported [56] that PI displayed antagonistic properties to activated ligand by the Toll-like receptors (TOR)—a crucial event for the virulence of certain viruses of the lung. Despite the absence of a quantitative analysis of the entire PI pool, it has been shown that erucic acid inhibits the infection of influenza A and H7N9 viruses [57,58]. Its preventive mode of action consists of down-regulating pro-inflammatory mediators, pro-apoptotic signaling, and aggravating immunological inflammation, underpinning its potential role in lung protection and disease prognosis management [53]. 

In liver PI, the nervonic acid proportion decreased as a result of FBs’ toxicity. A plausible mechanism behind decreased nervonic acid level is the translocation of acyl chains across bilayer lipid classes, which may alter hepato-signal transduction and membrane traffic. However, the present study did not investigate the related molecular signaling. Probably the most PI-lipid disintegration event was the proportional depletion of DHA (also occurred in lung-PI), which decreased the total sum of n-3 FAs and raised the n-6:n-3 ratio. [18] have reported a similar DHA’s finding in rat liver-PI. In our case, the decrease in DHA appears independent of feed intake and lipid peroxidation (no marked change was noticed; see Table 8). This finding with n-3 FA patterns in PC supports the proposal of [23] that FBs hamper Δ5D and Δ6D activities in hepato-microsomal membranes.

### 3.7. Antioxidant Enzymes and Lipid Peroxidation 

The lipid bilayers’ peroxidation is a complex chain reaction encompassing enzymatic (catalyzed by the lipoxygenase family) and/or non-enzymatic reactions (generation and propagation of reactive oxygen species (ROS), uptake of oxygen, and disruption of the double bond in unsaturated FAs). Thus, lipid peroxidation ultimately leads to the destruction of cellular biomolecules, including membrane lipids [59,60]. According to numerous in vivo and in vitro reports (reviewed by [61]), oxidative stress is one of the FBs’ mediated toxicity pathways. However, there is no full compliance whether oxidative stress is a direct or indirect route of the FBs-toxicity. According to [62], ROS generation is rather a consequence of FBs-toxicity than a direct toxicity event of FBs.

In this study, neither the low molecular weight antioxidant (GSH) nor the antioxidant enzyme (glutathione peroxidase (GPx)) of the glutathione redox system, or the end product of lipid peroxidation TBARS (expressed as malondialdehyde (MDA)) were altered in liver or lung tissues. It is important to highlight that the literature lacks data on the porcine pulmonary oxidative capacity under FBs exposure. However, pure doses of FB_1_ (eq. 20, 50, and 100 mg FB_1_/kg diet) were reported to induce lipid peroxidation in rat lungs, although not in a dose-dependent manner. Moreover, alterations in MDA levels varied among the time points—5 and 10 days of exposure [63]. Variation in oxidative findings may be attributable to the ceramide proportion within the tissue, in which the up-regulation of CerS has been associated with apoptosis and oxidative stress [64]. Anyhow, the compensatory re-modulation in the FA profile of investigated polar lipids likely assisted in the prevention of strong propagation of lipid peroxidation. 

Our findings in the liver are similar to those obtained at 5 mg FBs/kg diet for 3 weeks [16], but inconsistent with those of [17], when 20 mg FBs/kg diet for 10 days elevated the lipid peroxidation biomarker and triggered the enzymatic defense system. We speculate that the variation in outcomes may be due to the high SAT and MUFA levels in hepatic membrane lipid fractions, such as PC and PI. [19] have reported that high SAT and MUFA levels refer to rigid membranes, a cellular defense mechanism against the attack of free radicals. In addition, the elevated proportion of oleic acid in PC and PE fractions possibly exhibited antioxidant properties that neutralized the end product of lipid peroxidation. Oleic acid has been reported to augment GSH biosynthesis in murine liver [65]. The novelty of our study is that it confirms the proposals that lipid profile alterations in bilayer lipids by FBs are rather a re-modulation mechanism than a lipid peroxidation-consequence [16], and that elevation of SAT and MUFA may express a protective mechanism against FBs toxicity [19].

### 3.8. Histopathology 

Compared to studies performed on rats, there are fewer studies on porcine histological modifications induced by FBs exposure. Once, FBs target the swine lung and liver, and thus, histological modifications were expected as they have been reported in porcine tissues [8,17]. In the livers of piglets exposed to FBs, we observed a depletion in glycogen microvacuoles that was associated with a lower extent of cytoplasmic vacuolization. Furthermore, intoxicated animals exhibited scattered solitaire hepatocellular necrosis, swollen and scattered focal proliferation of MPS. These findings concur with the recently published findings of [8,17], demonstrating together the dose-dependent toxic effects of FBs on the liver. In contrast, our findings contradict those of [16] in piglets, where no liver lesions were identified. We attribute our detected hepatotoxicity to the FBs retention level and exposure period in the liver throughout the trial, since in the study of [16] a low FBs dose was administered. Notably, our hepato-histopathology was confirmed by alterations in serum biochemicals; 30 mg FBs/kg diet for 21 days elevated AST, ALP, and cholesterol concentrations (six-, five-, and two-folds, respectively; data not shown). The novelty of this study is that no marked lipid peroxidation was detected, strongly suggesting that the perpetuation of sphingolipids and hepatocellular lipids are the primary determinants of observed hepatotoxicity. 

Among all treatments, the lungs of some animals showed mild focal interstitial lympho-histiocytic infiltrations (seen interstitially), as well as mild focal fibrosis of the visceral pleura. These histological alterations may relate to the PPE progression. However, a single piglet developed PPE upon exposure to 30 mg FBs/kg feed, revealing alveoli filled with finely granular, pale eosinophilic serous fluid. This finding appeared to be consistent with the studies [9,66,67] reporting that FBs, especially FB_1_, are PPE inducers in swine. Events such as the blockage of L-type calcium channels and left ventricular hypertrophy in the heart have been established as mediators for PPE [9]. Anyhow, the majority of intoxicated piglets in our study did not develop PPE. According to [68], when weanling piglets fed FBs-contaminated diets in a dose response manner (175, 101, 39, 23, 5, and <1 mg FB_1_ + FB_2_/kg feed) for 14 days, only 175 mg FBs/kg feed induced PPE. Herein, we are rather aiming to present possible inconsistencies in results than establish that PPE development requires extremely high doses of FBs. Indeed, PPE has been observed in some weaned piglets upon exposure to 20 mg FB_1_/kg diet for 10 days [17]. On the other hand, in a recent study by [8], a relatively very low dose of FBs (3.7 mg FBs/kg diet) for 28 days exerted pathomorphological changes in the weaned piglet heart without developing PPE. In the present study, hyperaemia and lesions found in the lungs may either be mediators for PPE development or represent subclinical evidence of a latent airway infection or immunosuppression of the respiratory system (none was tested) that implicated FBs exposure. FB_1_ has been shown to exert an immunosuppressive effect in pigs exposed to relatively low level of 10 mg FB_1_/kg feed [69], which was reported to aggravate the progression of respiratory pathogenicity induced by *Mycoplasma hyopneumoniae* [32] and *Pasteurella multocida* [70]. However, further investigation in immunosuppression induction by FBs related to dose and exposure period are important. 

## 4. Conclusions

The study investigated the effects of FBs in a dose-dependent manner on the FA profiles of membrane lipids from the liver and lungs of piglets. This is the first study to report alterations in the FAs profiles of porcine lung phospholipid classes in response to FBs-exposure. The relatively high dose of FB_1_ had no marked effect on piglet production traits or oxidative stress markers. Only minor characteristic effects were detected in FA profiles of membrane lipid fractions. Alterations in linear dose-dependence were observed in liver PC, as well as lung PC and PS fractions. The obtained data revealed variations in tissue-specific membrane lipid responses. Furthermore, findings suggest alterations in enzymes involved in FA metabolism, such as elongase, Δ5D, Δ6D, and Δ9D. Disruption in these enzymes was characterized by the proportional depletion of polyunsaturated FAs and the augmentation of total monounsaturation. Alterations detected in membrane lipids are not assumed to be mainly responsible for liver and lung toxicities, rather than consequences/mediators of other events that might have led to tissues’ injuries. The study lacks the quantitative determination of ceramide and polar lipids, which could provide additional explanation for the results. Therefore, further studies investigating the quantitative effects of FBs on lipidomes of the liver and lungs are highly important. 

## 5. Materials and Methods

### 5.1. Experimental Design, Animals and Feeding

The study was a completely randomized design (CRD) that involved 18 weaned Danbred male piglets at the age of 35 days with semi-equal average body weights. Randomly, piglets were equally assigned to three treatments, with each group consisting of six animals. Each animal was kept individually in a metabolic cage with an area of 80 cm × 80 cm. In the beginning, all piglets underwent a 14-day adaptation period. After the adaptation period (at the exact age of 49 days), the initial body weight was determined for each animal individually. The duration of the feeding trial was 21 days; besides the control (FBs-free), one group was fed on a contaminated diet (15 mg FBs/kg; FB_1_, FB_2_, and FB_3_ in a fungal culture), whereas the other group was fed a high FBs-contaminated diet (30 mg total FBs/kg). 

The diet formulation and nutrient optimization were performed by Bonafarm-Bábolna Takarmány Kft (Bábolna, Hungary). The formulated diet was analyzed in accordance with [71], and its FA composition was determined (Table 9). Upon animals’ arrival at the experimental unit, the portion of feed offered was estimated based on the initial body weight of piglets, and in correspondence to the actual consumption, the daily offered diets were adjusted. The diet was offered twice a day, in equal proportions (equivalent to 0.5 kg feed consumption/animal/day during the first week and gradually increased to reach 0.7 kg feed intake/animal/day at the last week). At the end of each day, the feed residual was measured back for the daily feed intake determination. During the whole study, water was offered *ad libitum* to the piglets via automatic drinkers. The house environmental conditions (such as temperature and humidity) were adjusted by the needs of weaned piglets.

At the end of the trial, the piglet bodyweight was determined individually. Later on, the piglets were euthanized by exsanguination after sedation (Euthanyl-Pentobarbital Sodium, 400 mg/mL, Dechra Veterinary Products, Shrewsbury, UK), and their liver and lung were sampled for analysis. From the jugular vein, the fresh blood was collected into heparinized (20 IU/mL whole blood) tubes and was centrifuged for 10 min at 1000× *g* (SIGMA 3-30KS refrigerated centrifuge, Osterode am Harz, Germany) for plasma separation. Both plasma and liver samples were immediately stored at −80 °C for further analysis. 

### 5.2. Feed Mycotoxin Contamination

The fungal strain *Fusarium verticillioides* (MRC 826) was inoculated on pre-soaked, sterile maize kernels, in a form of spore suspension. The incubation was set to 25 °C for 5 weeks, and the final FBs concentration in dried culture material was harvested in different production batches. Details on fungal culture preparation were published earlier [72]. The final FB_1_ concentrations were 2000–4000 mg/kg in the air-dried culture material harvested in different batches. The FB_2_ concentration of the inoculum materials was ca. 30% of the FB_1_ content and the FB_3_ concentration was ca. 10–15% of the FB_1_ content. The fungal culture was mixed into the ration of the experimental animals so as to provide a daily FBs (FB_1_ + FB_2_ + FB_3_) feed concentration of 15 and 30 mg/kg. The diet fed to the control group did not contain detectable amounts of FBs, whereas the experimental diets were mixed with fungal culture to provide 15 and 30 mg FBs/kg diet. In the diets, the absence of FBs co-occurrence with deoxynivalenol (DON), zearalenone (ZEN), and T-2 toxin was also confirmed, in which the analyzed diets did not contain detectable concentrations (below the limit of detection; 0.053, 0.005, and 0.011 mg/kg for DON, ZEN, and T-2 toxin, respectively). The content of FBs was determined by the LC-MS-2020 mass spectrometer (Shimadzu, Kyoto, Japan) [73].

### 5.3. Fatty Acid Composition of Phospholipid Classes

The liver, lung (after frozen storage at −20 °C), and diet samples were homogenized (IKA T25 Digital Ultra Turrax, Staufen, Germany) in the 20-fold volume of chloroform:methanol (2:1), and the total lipid content (complex lipids) was extracted according to [74]. Solvents were ultrapure-grade (Merck Sigma-Aldrich, Schnelldorf, Germany) and 0.01% butylated hydroxytoluene was added to prevent FA oxidation.

In the case of the diet, the FAs were transmethylated by the base-catalyzed sodium-methoxide method of [75]. For the separation of phospholipid classes in liver and lungs, thin layer chromatography (TLC) was used. Extracted complex lipids were spotted onto pre-dried (110 °C, 2 h) 20 cm × 20 cm TLC plates (Sigma Cat. No.: 99570). The separation was performed in one dimension, using the eluent mixture of methyl acetate–isopropanol–chloroform–methanol–aqueous 0.025% KCl (25:25:25:10:9, *v*/*v*/*v*/*v*/*v*), developing the plate until the top, in an all-glass, covered TLC chamber [76]. Primuline spray (5 mg in 100 mL of acetone:water (80:20, Merck-Sigma Cat. No.: 206865, Schnelldorf, Germany)) was used to stain lipid spots, and detection was performed under ultraviolet light (365 nm). To identify PL classes, certified reference materials were used as follows: N-Acyl-D-sphingosine-1-phosphocholine (Merck-Sigma Cat. No. S0756), L-α-phosphatidylcholine (Merck-Sigma Cat. No.: P3556), L-α-phosphatidylethanolamine (Merck-Sigma Cat. No.: P7943), L-α-phosphatidylserine (Merck-Sigma Cat. No. 870336C), and L-α-phosphatidylinositol (from Glycine max, Merck-Sigma Cat. No. P6636). Identified fractions were scraped off from the plates and were extracted 3 times into the TLC eluent mixture. Subsequently, the solvent was evaporated entirely and lipids were trans-methylated with an acid-catalysed method, using 1% H_2_SO_4_ in methanol [77]. Fatty acid methyl esters were extracted into 150 μL ultrapure n-hexane for gas chromatography, which was performed on a GC-Shimadzu 2030 equipped with an AOC 20i automatic injector (Kyoto, Japan), a Phenomenex Zebron ZB-WAXplus capillary GC column (30 m × 0.25 mm ID, 0.25 μm film, Phenomenex Inc., Torrance, CA, USA) and a flame ionization detector (FID) detector. Characteristic operating conditions were: injector temperature: 220 °C; detector temperature: 250 °C; helium flow: 28 cm/sec. The oven temperature was graded: from 60 (2 min hold) to 150 °C, from 150 to 180 °C: 2 °C/min and 10 min at 180 °C, from 180 to 220 °C: 2 °C/min, and 16 min at 220 °C. The makeup gas was nitrogen. 

To identify individual FAs, an authentic external FA standard mixture (Merck Sigma-Aldrich, Schnelldorf, Germany) was used. Fatty acid results were expressed as weight % of total FA methyl esters. The unsaturation index (UI) was calculated to express the number of double bonds in 100 FA chains. Calculation was performed with the LabSolutions 5.93 software, using the PostRun module (Shimadzu, Kyoto, Japan) with manual peak integration.

### 5.4. Antioxidant Status and Lipid Peroxidation

Samples of lung and liver were stored at −82 °C before analysis. Lipid peroxidation was assessed with the determination of thiobarbituric acid reactive substances and expressed as malondialdehyde, which was served as standard [78] in the 10-fold volume of tissue homogenate in physiological saline (0.65 *w/v*% NaCl). The amount of GSH and GPx activity was measured in the 10,000× *g* supernatant fraction of tissue homogenate. The quantification of the GSH was performed according to the method of [79], using 5,5′-dithiobis-2-nitrobenzoic acid (DTNB) as a sulfhydryl reagent to form a yellow derivative, which is measurable at 412 nm. The activity of GPx was determined according to [80] applying an end-point direct assay with GSH and cumene hydroperoxide as co-substrates.

GSH concentration and GPx activity were calculated from the protein content of the 10,000× *g* supernatant fraction after centrifugation (10 min at 4 °C), which was measured by the Folin-phenol reagent [81]. In all instances, the color was measured with UV–Vis spectrophotometry in 10 mm pathway optical glass cuvettes.

### 5.5. Histological Preperation and Assessment

Tissue samples of the liver and lung were stored in 10% neutrally buffered formalin and embedded into paraffin. For light microscopic analysis, microtome slides of 5 micrometers were prepared and stained with hematoxyllin–eosin. Detected lesions in the liver were decreased glycogen content of liver cells, hepatocellular necrosis, and swelling and proliferation of MPS cells. In the lung, lesions included interstitial lymphohistiocytic infiltration and pleural fibrosis. The main pathological alterations have been described and scored according to their extent and severity as follows: 0 = no alteration, 1 = slight/small scale/few, 2 = medium degree/medium scale/medium number, 3 = pronounced/extensive/numerous. Images were taken on an Olympus BX43 microscope with a DP23 camera (Olympus, Tokyo, Japan).

Histopathological examination was performed in accordance with the Decree No. 9/2001 (03.30) of the Ministry of Health and the Ministry of Agriculture and Rural Development and the guidelines of the OECD Good Laboratory Practice for Chemicals [82].

### 5.6. Statistical Analysis

All data were tested for normality (Shapiro–Wilk test), whereas the extent of standard deviation was compared between groups with Levene’s F test. After this, the univariate analysis of variance (ANOVA) was used on the control and total FB-fed group means, with the Least Significant Difference (LSD) “post hoc” test for detailed inter-group differences. For dose–response determination, the Pearson correlation was calculated between offered doses of FBs and further FA variables, always using individual data-pairs. Identified *p*-values > 0.05 were subjected to linear regression analysis, whereas only R^2^ greater than 0.6 are reported. The software that performed data evaluation was IBM SPSS 20 [83]. For the significance level identification, the calculated probability of a *p*-value < 0.05 was set for all tests.

Principal Component Analysis (PCA) was performed on the FA profile of the different phospholipids from liver and lungs with the Unscrambler 9.7. software [84] to seek principal components describing the variance responsible for the “group formation” with the highest possible efficacy. The sole purpose of PCA was not to discriminate between certain groups of treatments based on their chemical composition, but rather to describe the basic orientation of the groups within the multidimensional space described by the variables investigated (e.g., FA profile elements). The orientation of the samples is described by the score plot, which shows the scores of each sample along with the first two principal components. The variable impact is presented with the loadings bar graph, which shows the contribution of the variance of each investigated variable to the full variance of the first principal component; that is, the values of the loadings graph are the weights for each original variable when calculating the principal component.

## Figures and Tables

**Table 1:** The growth performance (in g), absolute and relative organ weights (g and %, respectively), and feed conversion efficiency of piglets (n = 6 animals/treatment). The results represent mean ± standard deviation (SD).

Parameter	Control	15 mg FBs	30 mg FBs
Initial body weight	12,980 ± 1720	13,800 ± 1200	13,800 ± 1080
Final body weight	21,467 ± 1735	23,067 ± 1454	23,367 ± 629
Body weight gain	8483 ± 2406	9267 ± 2240	9567 ± 1181
Cumulative feed intake	19,759 ± 2102	20,450 ± 1352	20,382 ± 1353
Feed conversion efficiency	2069 ± 299	2230 ± 246	2455 ± 389
Absolute liver weight	527.4 ± 42.1	587.2 ± 56.9	563.0 ± 69.2
Absolute lung weight	227.2 ± 45.0	237.1 ± 43.0	253.0 ± 70.0
Relative liver weight (%)	2.48 ± 0.38	2.55 ± 0.23	2.42 ± 0.45
Relative lung weight (%)	1.06 ± 0.20	1.03 ± 0.15	1.09 ± 0.30

**Table 2:** Fatty acid profiles of sphingomyelins from liver and lungs of the experimental piglets (n = 6 animals/treatment). The results represent mean ± standard deviation (SD).

Fatty Acid	Control	15 mg FBs	30 mg FBs	Control	15 mg FBs	30 mg FBs
	Liver			Lung		
C12:0	0.06 ± 0.04 ^a^	0.09 ± 0.03 ^ab^	0.10 ± 0.03 ^b^	0.12 ± 0.09 ^ab^	0.05 ± 0.05 ^a^	0.20 ± 0.03 ^b^
C14:0	0.69 ± 0.08	0.61 ± 0.16	0.61 ± 0.15	1.27 ± 0.34 ^a^	1.36 ± 0.56 ^ab^	1.96 ± 0.69 ^b^
C16:0	34.7 ± 4.01	34.0 ± 3.87	30.7 ± 5.21	51.3 ± 2.77	47.3 ± 3.87	47.9 ± 6.09
C16:1n7	- ± -	- ± -	- ± -	0.34 ± 0.26	0.59 ± 0.32	0.60 ± 0.33
C18:0	29.1 ± 5.53	30.9 ± 2.05	26.3 ± 4.81	18.4 ± 1.73	16.1 ± 2.84	15.5 ± 2.84
C18:1n9c	5.27 ± 1.91	4.43 ± 1.71	5.5 ± 2.46	2.00 ± 1.29	2.59 ± 1.16	3.26 ± 1.45
C18:1n7	0.74 ± 0.35	0.57 ± 0.21	0.84 ± 0.34	0.41 ± 0.28	0.52 ± 0.28	0.63 ± 0.33
C18:2n6	3.26 ± 1.58	2.92 ± 1.22	3.95 ± 2.27	0.99 ± 0.71	1.26 ± 0.70	1.55 ± 0.87
C20:0	1.53 ± 0.25 ^b^	1.46 ± 0.27 ^b^	1.09 ± 0.36 ^a^	1.91 ± 0.24	2.20 ± 0.54	1.90 ± 0.37
C20:1n9	- ± -	- ± -	- ± -	0.23 ± 0.05	0.20 ± 0.02	0.20 ± 0.05
C20:4n6	0.91 ± 0.60	1.36 ± 0.66	1.31 ± 0.54	0.76 ± 0.65	0.86 ± 0.44	1.12 ± 0.64
C22:0	1.60 ± 0.23 ^a^	1.85 ± 0.27 ^ab^	2.20 ± 0.58 ^b^	2.95 ± 0.55	3.74 ± 1.08	3.38 ± 0.61
C22:1n9	9.49 ± 3.13	10.8 ± 3.76	11.4 ± 4.37	5.62 ± 1.55 ^a^	6.72 ± 1.1 ^ab^	8.97 ± 2.71 ^b^
C23:0	1.42 ± 0.85	1.44 ± 0.36	1.73 ± 1.11	0.57 ± 0.07	0.73 ± 0.06	0.67 ± 0.24
C24:0	5.10 ± 3.58	4.95 ± 1.14	6.26 ± 2.77	4.57 ± 0.86 ^b^	5.07 ± 1.07 ^b^	3.28 ± 0.58 ^a^
C24:1n9	3.77 ± 2.85	2.81 ± 0.95	4.71 ± 1.81	8.80 ± 2.01	10.8 ± 2.35	8.83 ± 2.00
saturation	72.8 ± 10.98	75.3 ± 4.90	68.6 ± 7.65	81.1 ± 2.75 ^b^	76.6 ± 1.24 ^a^	74.8 ± 3.39 ^a^
unsaturation	23.4 ± 3.65	22.0 ± 3.74	25.7 ± 6.54	18.9 ± 2.75 ^a^	23.4 ± 1.24 ^b^	25.2 ± 3.39 ^b^
monounsaturation	19.3 ± 3.81	17.7 ± 4.42	20.5 ± 7.81	17.3 ± 2.36 ^a^	21.3 ± 1.51 ^b^	22.5 ± 2.89 ^b^
polyunsaturation	4.17 ± 1.77	4.28 ± 1.65	5.27 ± 2.79	1.62 ± 1.32	2.12 ± 1.13	2.67 ± 1.42
n-6	4.17 ± 1.77	4.28 ± 1.65	5.27 ± 2.79	1.62 ± 1.32	2.12 ± 1.13	2.67 ± 1.42
odd chain FA	1.42 ± 0.85	1.44 ± 0.36	1.73 ± 1.11	0.57 ± 0.07	0.73 ± 0.06	0.67 ± 0.24
unsaturation index	29.4 ± 4.5	29.0 ± 3.84	33.6 ± 6.65	21.8 ± 4.80 ^a^	27.3 ± 2.50 ^ab^	30.1 ± 5.54 ^b^
average chain length	17.6 ± 1.59	17.9 ± 1.18	17.6 ± 1.52	18.1 ± 0.29	18.5 ± 0.34	18.3 ± 0.41

n-6, omega-6; FA, fatty acid; -, fatty acid below the detection limit or not possible to calculate due to a fatty acid limitation in samples; ^a, b^ values with different letters refer to a significant difference among the treatments (*p* < 0.05).

**Table 3:** Fatty acid profile of phosphatidylcholines from liver and lungs of the experimental piglets (n = 6 animals/treatment). The results represent mean ± standard deviation (SD).

Fatty Acid	Control	15 mg FBs	30 mg FBs	Control	15 mg FBs	30 mg FBs
	Liver			Lung		
C12:0	0.01 ± -	- ± -	- ± -	0.02 ± 0.01 ^ab^	0.01 ± 0.00 ^a^	0.03 ± 0.01 ^b^
C14:0	0.21 ± 0.02 ^a^	0.30 ± 0.10 ^b^	0.37 ± 0.05 ^b^	2.99 ± 0.68 ^a^	3.13 ± 0.41 ^a^	4.08 ± 0.65 ^b^
C16:0	22.0 ± 0.83	21.8 ± 1.99	23.9 ± 2.68	61.9 ± 1.28 ^a^	66.1 ± 2.93 ^b^	61.1 ± 1.58 ^a^
C16:1n7	0.55 ± 0.06 ^a^	0.64 ± 0.06 ^b^	0.79 ± 0.08 ^c^	1.98 ± 0.42	1.67 ± 0.59	2.27 ± 0.64
C18:0	28.1 ± 1.84 ^b^	28.6 ± 1.51 ^b^	26.2 ± 1.67 ^a^	9.42 ± 0.88 ^b^	9.42 ± 1.34 ^b^	7.35 ± 1.12 ^a^
C18:1n9	12.4 ± 1.89 ^a^	14.1 ± 1.42 ^a^	17.1 ± 1.12 ^b^	8.68 ± 0.76 ^a^	7.54 ± 1.26 ^a^	9.99 ± 0.68 ^b^
C18:1n7	1.96 ± 0.21	1.90 ± 0.30	1.95 ± 0.13	2.31 ± 0.21 ^b^	1.75 ± 0.37 ^a^	1.93 ± 0.27 ^a^
C18:2n6	13.8 ± 0.96 ^b^	14.0 ± 1.36 ^b^	12.0 ± 1.04 ^a^	5.60 ± 0.31 ^ab^	4.73 ± 1.01 ^a^	6.21 ± 1.21 ^b^
C18:3n6	0.17 ± 0.04	0.19 ± 0.03	0.17 ± 0.02	0.13 ± 0.04 ^b^	0.07 ± 0.02 ^a^	0.09 ± 0.03 ^ab^
C18:3n3	0.14 ± 0.03 ^a^	0.17 ± 0.02 ^b^	0.14 ± 0.02 ^a^	0.05 ± 0.01	0.04 ± 0.01	0.05 ± 0.02
C20:0	0.07 ± 0.01	0.07 ± 0.02	0.06 ± 0.01	0.09 ± 0.03	0.10 ± 0.04	0.09 ± 0.01
C20:1n9	0.11 ± 0.02	0.12 ± 0.03	0.11 ± 0.03	0.15 ± 0.10	0.12 ± 0.03	0.13 ± 0.05
C20:2n6	0.18 ± 0.06	0.17 ± 0.05	0.13 ± 0.02	0.19 ± 0.05 ^a^	0.12 ± 0.05 ^b^	0.13 ± 0.02 ^b^
C20:3n6	0.46 ± 0.12	0.49 ± 0.04	0.47 ± 0.05	0.44 ± 0.04 ^b^	0.34 ± 0.08 ^a^	0.44 ± 0.08 ^b^
C20:4n6	15.2 ± 2.20 ^b^	13.7 ± 1.35 ^ab^	12.7 ± 2.69 ^a^	3.95 ± 0.36 ^b^	2.97 ± 0.63 ^a^	3.49 ± 0.58 ^ab^
C20:5n3	0.26 ± 0.09	0.29 ± 0.11	0.22 ± 0.04	0.05 ± 0.01	0.05 ± 0.01	0.05 ± 0.01
C22:0	- ± -	- ± -	- ± -	0.07 ± 0.02	0.10 ± 0.04	0.08 ± 0.02
C22:1n9	1.19 ± 0.47	1.25 ± 0.67	1.15 ± 0.41	1.49 ± 0.17 ^a^	1.23 ± 0.37 ^a^	2.07 ± 0.41 ^b^
C22:5n3	1.22 ± 0.23 ^b^	0.91 ± 0.13 ^a^	0.96 ± 0.24 ^ab^	0.13 ± 0.02 ^b^	0.10 ± 0.03 ^a^	0.10 ± 0.02 ^a^
C24:0	0.07 ± 0.03	0.05 ± 0.04	0.14 ± 0.06	0.08 ± 0.02 ^ab^	0.14 ± 0.08 ^b^	0.06 ± 0.02 ^a^
C22:6n3	1.82 ± 0.60	1.20 ± 0.24	1.42 ± 0.69	0.16 ± 0.03 ^b^	0.07 ± 0.04 ^a^	0.04 ± 0.02 ^a^
C24:1n9	- ± -	0.06 ± -	0.17 ± 0.10	0.11 ± 0.04	0.22 ± 0.12	0.22 ± 0.10
saturation	50.5 ± 2.11	50.9 ± 2.76	50.6 ± 4.25	74.6 ± 1.76 ^a^	79.0 ± 3.77 ^b^	72.8 ± 1.58 ^a^
unsaturation	49.5 ± 2.11	49.0 ± 2.73	49.4 ± 4.25	25.4 ± 1.76 ^b^	21.0 ± 3.77 ^a^	27.2 ± 1.53 ^b^
monounsaturation	16.2 ± 1.99 ^a^	18.0 ± 1.83 ^a^	21.1 ± 1.03 ^b^	14.7 ± 1.15 ^b^	12.5 ± 2.18 ^a^	16.6 ± 0.57 ^c^
polyunsaturation	33.3 ± 3.55 ^b^	31.1 ± 2.73 ^ab^	28.2 ± 4.23 ^a^	10.7 ± 0.69 ^b^	8.47 ± 1.75 ^a^	10.6 ± 1.30 ^b^
n-3	3.44 ± 0.75 ^b^	2.52 ± 0.34 ^a^	2.75 ± 0.96 ^ab^	0.38 ± 0.04 ^b^	0.25 ± 0.03 ^a^	0.23 ± 0.03 ^a^
n-6	29.9 ± 2.81 ^b^	28.6 ± 2.58 ^ab^	25.5 ± 3.39 ^a^	10.3 ± 0.69 ^b^	8.22 ± 1.74 ^a^	10.4 ± 1.29 ^b^
n-6:n-3	8.98 ± 1.75 ^ab^	11.5 ± 1.49 ^b^	9.8 ± 1.92 ^a^	27.2 ± 3.91 ^a^	33.29 ± 7.33 ^a^	44.5 ± 7.01 ^b^
unsaturation index	124 ± 12.5	115 ± 7.94	112 ± 16.9	45.6 ± 3.08 ^b^	36.3 ± 6.71 ^a^	45.7 ± 3.53 ^b^
average chain length	18.0 ± 0.09	18.0 ± 0.06	17.9 ± 0.17	16.8 ± 0.05	16.7 ± 0.08	16.8 ± 0.08

n-3, omega-3; n-6, omega-6; n-6:n-3, ratio of omega-6 to omega-3; -, fatty acid below the detection limit or not possible to calculate due to a fatty acid limitation in samples; ^a, b^ values with different letters refer to a significant difference among the treatments (*p* < 0.05).

**Table 4:** Parameters of the linear dose response equations of fatty acids in the different phospholipid classes, obtained from liver and lung. Data represent only cases with R^2^ above 0.6.

Liver				Lung			
Parameter	Slope	Constant	R^2^	Parameter	Slope	Constant	R^2^
Phosphatidylcholine				Phosphatidylcholine			
C16:1	−44.129	89.188	0.69	C22:6n3	31.164	−178.028	0.705
C18:1n9	−44.357	4.073	0.633	n-3	53.773	−133.946	0.667
monousaturation	−53.704	3.710	0.609	n-6:n-3	−20.943	1.027	0.594
				**Phosphatidylserine**			
				C22:0	40.488	−52.0917	0.782
				C24:0	40.566	−134.894	0.645

n-3, omega-3; n-6:n-3, omega-6 to omega-6 ratio.

**Table 5:** Fatty acid profile of phosphatidylethanolamines from liver and lungs of the experimental piglets (n = 6 animals/treatment). The results represent mean ± standard deviation (SD).

Fatty Acid	Control	15 mg FBs	30 mg FBs	Control	15 mg FBs	30 mg FBs
	Liver			Lung		
C12:0	0.02 ± 0.00	0.03 ± 0.01	0.02 ± 0.00	0.07 ± 0.03 ^ab^	0.03 ± 0.01 ^a^	0.09 ± 0.03 ^b^
C14:0	0.14 ± 0.03	0.14 ± 0.04	0.12 ± 0.02	0.30 ± 0.17	0.36 ± 0.24	0.40 ± 0.11
C16:0	11.9 ± 1.33	11.3 ± 1.52	11.5 ± 1.51	18.9 ± 2.85	19.3 ± 6.07	20.2 ± 3.23
C16:1n7	0.16 ± 0.03 ^a^	0.20 ± 0.03 ^b^	0.20 ± 0.04 ^b^	0.41 ± 0.12 ^a^	0.56 ± 0.10 ^ab^	0.73 ± 0.24 ^b^
C18:0	36.5 ± 1.24	37.2 ± 1.94	39.4 ± 5.11	21.7 ± 1.49	19.9 ± 0.79	20.7 ± 2.71
C18:1n9	5.49 ± 0.56 ^a^	6.27 ± 1.33 ^ab^	7.11 ± 1.08 ^b^	14.3 ± 0.66	15.2 ± 1.01	15.3 ± 0.73
C18:1n7	1.06 ± 0.11	1.00 ± 0.23	1.01 ± 0.18	2.57 ± 0.46	2.72 ± 0.29	2.70 ± 0.20
C18:2n6	6.72 ± 0.80	7.19 ± 0.62	6.33 ± 0.83	5.47 ± 0.41 ^a^	6.31 ± 0.49 ^a^	7.36 ± 1.07 ^b^
C18:3n6	- ± -	- ± -	- ± -	0.21 ± 0.17	0.19 ± 0.11	0.07 ± -
C18:3n3	- ± -	- ± -	- ± -	0.12 ± -	0.13 ± 0.06	0.12 ± 0.03
C20:0	0.09 ± 0.01	0.07 ± 0.01	0.06 ± 0.04	0.16 ± 0.03	0.13 ± 0.01	0.16 ± 0.04
C20:1n9	0.15 ± 0.03	0.14 ± 0.03	0.12 ± 0.03	0.40 ± 0.04	0.39 ± 0.14	0.42 ± 0.08
C20:2n6	0.15 ± 0.04	0.11 ± 0.06	0.09 ± 0.04	0.17 ± 0.08	0.19 ± 0.13	0.16 ± 0.10
C20:3n6	0.33 ± 0.03	0.36 ± 0.07	0.33 ± 0.05	1.05 ± 0.08	1.21 ± 0.25	1.19 ± 0.19
C20:4n6	28.3 ± 1.84	27.9 ± 1.75	26.0 ± 3.04	28.3 ± 2.08	27.5 ± 3.94	24.0 ± 4.69
C20:5n3	0.26 ± 0.08 ^ab^	0.36 ± 0.15 ^b^	0.17 ± 0.06 ^a^	0.23 ± 0.03	0.33 ± 0.11	0.28 ± 0.08
C22:0	- ± -	- ± -	- ± -	0.07 ± 0.01 ^b^	0.04 ± 0.01 ^a^	0.04 ± 0.01 ^a^
C22:1n9	2.01 ± 0.36	2.04 ± 0.42	1.71 ± 0.47	3.06 ± 0.62 ^a^	2.84 ± 0.42 ^a^	4.14 ± 1.03 ^b^
C22:5n3	2.29 ± 0.29	1.94 ± 0.15	1.90 ± 0.43	1.76 ± 0.18 ^ab^	1.85 ± 0.41 ^b^	1.48 ± 0.24 ^a^
C22:6n3	4.46 ± 0.90	3.70 ± 0.88	3.65 ± 0.90	1.01 ± 0.33	1.01 ± 0.36	0.73 ± 0.34
saturation	48.6 ± 1.65	48.8 ± 2.44	51.0 ± 6.40	41.2 ± 1.98	39.7 ± 5.75	41.6 ± 5.00
unsaturation	51.4 ± 1.65	51.2 ± 2.45	48.5 ± 6.02	58.8 ± 1.98	60.3 ± 5.75	58.4 ± 5.00
monounsaturation	8.87 ± 0.75	9.65 ± 1.86	9.91 ± 1.23	20.69 ± 0.51 ^a^	21.7 ± 1.20 ^ab^	23.24 ± 1.76 ^b^
polyunsaturation	42.5 ± 2.07	41.6 ± 2.44	38.5 ± 4.89	38.1 ± 1.94	38.5 ± 4.78	35.2 ± 4.07
n-3	7.01 ± 0.57 ^b^	6.00 ± 0.83 ^ab^	5.73 ± 1.03 ^a^	3.02 ± 0.25	3.23 ± 0.51	2.53 ± 0.33
n-6	35.5 ± 1.63	35.6 ± 1.76	32.8 ± 4.02	35.1 ± 1.89	35.3 ± 4.41	32.7 ± 3.85
n-6:n-3	5.08 ± 0.30 ^a^	6.01 ± 0.67 ^b^	5.69 ± 0.56 ^ab^	11.7 ± 1.09 ^ab^	11.0 ± 1.25 ^a^	13.0 ± 1.36 ^b^
unsaturation index	176 ± 9.69	171 ± 10.1	160 ± 21.1	156 ± 8.56	156 ± 17.9	144 ± 18.1
average chain length	18.8 ± 0.17 ^b^	18.7 ± 0.18 ^ab^	18.4 ± 0.32 ^a^	18.4 ± 0.11	18.4 ± 0.23	18.3 ± 0.19

n-3, omega-3; n-6, omega-6; n-6:n-3, ratio of omega-6 to omega-3, fatty acid; -, fatty acid below the detection limit or not possible to calculate due to a fatty acid limitation in samples; ^a, b^ values with different letters refer to a significant difference among the treatments (*p* < 0.05).

**Table 6:** Fatty acid profiles of phosphatidylserines from liver and lungs of the experimental piglets (n = 6 animals/treatment). The results represent mean ± standard deviation (SD).

Fatty Acid	Control	15 mg FBs	30 mg FBs	Control	15 mg FBs	30 mg FBs
	Liver			Lung		
C12:0	0.02 ± 0.01 ^a^	0.08 ± 0.02 ^b^	0.09 ± 0.02 ^b^	- ± -	- ± -	- ± -
C14:0	0.56 ± 0.13	0.66 ± 0.11	0.66 ± 0.09	0.39 ± 0.11	0.45 ± 0.18	0.58 ± 0.13
C16:0	26.8 ± 3.02	28.3 ± 4.08	29.2 ± 3.29	19.4 ± 2.49	18.8 ± 2.65	20.7 ± 3.29
C16:1n7	- ± -	- ± -	- ± -	0.18 ± 0.07	0.25 ± 0.12	0.32 ± 0.20
C18:0	41.8 ± 3.98	40.0 ± 2.54	38.1 ± 2.56	41.7 ± 1.98 ^b^	38.5 ± 2.05 ^a^	37.2 ± 1.83 ^a^
C18:1n9	5.81 ± 1.39	4.41 ± 1.34	6.58 ± 2.25	23.2 ± 2.11 ^ab^	24.6 ± 1.69 ^b^	21.9 ± 1.62 ^a^
C18:1n7	0.43 ± 0.11	0.46 ± 0.08	0.59 ± 0.16	1.15 ± 0.09	1.29 ± 0.11	1.18 ± 0.13
C18:2n6	1.73 ± 0.34	1.74 ± 0.28	2.08 ± 0.34	2.65 ± 0.25 ^a^	3.57 ± 0.29 ^b^	3.49 ± 0.78 ^b^
C18:3n6	- ± -	- ± -	- ± -	0.16 ± 0.05 ^a^	0.27 ± 0.10 ^b^	0.10 ± 0.02 ^a^
C20:0	0.32 ± 0.02	0.38 ± 0.09	0.41 ± 0.08	0.53 ± 0.06 ^b^	0.44 ± 0.04 ^a^	0.41 ± 0.06 ^a^
C20:1n9	0.36 ± 0.05 ^b^	0.23 ± 0.01 ^a^	0.27 ± 0.05 ^a^	0.37 ± 0.10	0.41 ± 0.03	0.40 ± 0.06
C20:3n6	0.43 ± 0.12	0.48 ± 0.10	0.57 ± 0.14	0.89 ± 0.10 ^a^	1.21 ± 0.24 ^b^	1.18 ± 0.17 ^b^
C20:4n6	8.39 ± 1.82	6.96 ± 2.12	7.50 ± 1.23	2.24 ± 0.34 ^a^	2.74 ± 0.40 ^b^	2.69 ± 0.20 ^b^
C22:0	- ± -	- ± -	- ± -	0.70 ± 0.17 ^b^	0.52 ± 0.06 ^a^	0.25 ± 0.04 ^a^
C22:1n9	9.30 ± 1.54	11.7 ± 1.41	10.7 ± 3.00	5.46 ± 0.78 ^a^	6.05 ± 1.12 ^a^	8.79 ± 1.68 ^b^
C22:5n3	0.84 ± 0.11 ^b^	0.75 ± 0.16 ^ab^	0.66 ± 0.11 ^a^	0.35 ± 0.05	0.40 ± 0.04	0.35 ± 0.06
C24:0	0.13 ± 0.04	0.18 ± 0.07	- ± -	0.27 ± 0.04 ^b^	0.16 ± 0.05 ^a^	0.13 ± 0.04 ^a^
C22:6n3	2.04 ± 0.71	1.58 ± 0.83	1.22 ± 0.75	0.17 ± 0.04 ^b^	0.12 ± 0.01 ^a^	0.12 ± 0.03 ^a^
C24:1n9	0.08 ± 0.08 ^a^	0.38 ± 0.12 ^b^	0.28 ± 0.10 ^b^	0.22 ± 0.05	0.27 ± 0.12	0.24 ± 0.04
saturation	69.7 ± 5.21	69.4 ± 5.64	68.5 ± 4.10	63.0 ± 2.59 ^b^	58.8 ± 0.75 ^a^	59.1 ± 2.32 ^a^
unsaturation	27.5 ± 4.05	24.6 ± 6.17	28.2 ± 3.96	37.0 ± 2.59 ^a^	41.2 ± 0.74 ^b^	40.7 ± 2.56 ^b^
monounsaturation	15.8 ± 2.30	14.8 ± 6.54	18.3 ± 2.61	30.6 ± 2.62	32.8 ± 0.90	32.8 ± 2.75
polyunsaturation	13.1 ± 2.29	11.5 ± 2.97	11.6 ± 2.34	6.45 ± 0.51 ^a^	8.32 ± 0.88 ^b^	7.86 ± 1.13 ^b^
n-3	2.87 ± 0.72	2.33 ± 0.97	1.88 ± 0.84	0.53 ± 0.06	0.53 ± 0.04	0.46 ± 0.05
n-6	10.2 ± 1.65	9.18 ± 2.08	9.74 ± 1.59	5.92 ± 0.54 ^a^	7.80 ± 0.86 ^b^	7.39 ± 1.10 ^b^
n-6:n-3	3.63 ± 0.52 ^a^	4.16 ± 0.78 ^ab^	5.98 ± 2.46 ^b^	11.5 ± 2.02 ^a^	14.9 ± 1.42 ^b^	16.0 ± 1.96 ^b^
unsaturation index	69.8 ± 11.7	60.8 ± 13.3	63.9 ± 11.6	50.7 ± 3.04	58.1 ± 2.36	47.4 ± 23.4
average chain length	17.9 ± 0.57	17.3 ± 1.86	17.8 ± 0.74	18.0 ± 0.07	18.0 ± 0.07	24.3 ± 15.3

n-3, omega-3; n-6, omega-6; n-6:n-3, ratio of omega-6 to omega-3, fatty acid; -, fatty acid below the detection limit or not possible to calculate due to a fatty acid limitation in samples; ^a, b^ values with different letters refer to a significant difference among the treatments (*p* < 0.05).

**Table 7:** Fatty acid profiles of phosphatidylinositols from liver and lungs of the experimental piglets (n = 6 animals/treatment). The results represent mean ± standard deviation (SD).

Fatty Acid	Control	15 mg FBs	30 mg FBs	Control	15 mg FBs	30 mg FBs
	Liver			Lung		
C12:0	0.08 ± 0.01 ^a^	0.12 ± 0.02 ^b^	0.09 ± 0.01 ^ab^	- ± -	- ± -	- ± -
C14:0	0.38 ± 0.13	0.47 ± 0.11	0.36 ± 0.09	0.47 ± 0.06 ^b^	0.38 ± 0.05 ^a^	0.51 ± 0.05 ^b^
C16:0	17.8 ± 1.96	21.4 ± 4.82	16.7 ± 4.36	31.0 ± 1.57	30.5 ± 1.94	29.7 ± 3.49
C16:1n7	- ± -	- ± -	- ± -	0.94 ± 0.44	1.23 ± 0.55	0.87 ± 0.15
C18:0	39.9 ± 2.54 ^a^	44.7 ± 1.48 ^b^	42.8 ± 3.34 ^ab^	29.2 ± 2.15 ^b^	27.2 ± 2.32 ^ab^	26.6 ± 1.24 ^a^
C18:1n9	1.74 ± 0.72	1.56 ± 0.35	1.88 ± 0.41	10.3 ± 1.68	12.2 ± 2.27	12.0 ± 0.91
C18:1n7	0.23 ± 0.08	0.27 ± 0.08	0.26 ± 0.03	1.81 ± 0.25	2.01 ± 0.27	1.88 ± 0.15
C18:2n6	2.24 ± 0.41	2.25 ± 0.34	1.91 ± 0.34	5.10 ± 0.97	5.75 ± 1.08	6.62 ± 2.46
C20:3n6	0.48 ± 0.05	0.51 ± 0.17	0.58 ± 0.07	0.42 ± 0.53	0.49 ± 0.29	0.04 ± -
C18:3n3	- ± -	- ± -	- ± -	0.28 ± 0.21	0.21 ± 0.11	0.14 ± 0.05
C20:0	- ± -	- ± -	- ± -	0.18 ± 0.09	0.15 ± 0.06	0.19 ± 0.02
C20:1n9	- ± -	- ± -	- ± -	0.23 ± 0.07	0.21 ± 0.05	0.22 ± 0.03
C20:2n6	- ± -	- ± -	- ± -	0.07 ± 0.01	0.05 ± 0.02	0.06 ± 0.04
C20:3n6	- ± -	- ± -	- ± -	0.64 ± 0.07	0.67 ± 0.13	0.66 ± 0.12
C20:4n6	19.7 ± 1.68	19.5 ± 4.83	22.4 ± 2.35	12.8 ± 1.12	13.1 ± 1.50	11.3 ± 1.73
C22:0	- ± -	- ± -	- ± -	0.06 ± 0.02	0.03 ± 0.01	0.06 ± 0.05
C22:1n9	14.5 ± 5.42 ^b^	8.01 ± 1.76 ^a^	11.9 ± 5.67 ^ab^	6.01 ± 1.84 ^a^	5.39 ± 1.38 ^a^	8.55 ± 1.22 ^b^
C22:5n3	1.20 ± 0.30 ^b^	0.64 ± 0.14 ^a^	0.78 ± 0.34 ^a^	0.41 ± 0.05	0.42 ± 0.07	0.40 ± 0.06
C24:0	0.19 ± 0.05	0.12 ± 0.02	0.11 ± 0.03	- ± -	- ± -	- ± -
C22:6n3	0.58 ± 0.14 ^b^	0.36 ± 0.12 ^a^	0.31 ± 0.16 ^a^	0.19 ± 0.02 ^b^	0.15 ± 0.04 ^ab^	0.13 ± 0.08 ^a^
C24:1n9	0.50 ± 0.25 ^b^	0.17 ± 0.04 ^a^	0.13 ± 0.04 ^a^	- ± -	- ± -	- ± -
saturation	58.2 ± 3.91 ^a^	66.8 ± 4.4 ^b^	60.0 ± 7.75 ^ab^	60.9 ± 2.25 ^b^	58.3 ± 2.67 ^ab^	56.9 ± 2.91 ^a^
unsaturation	40.5 ± 4.40 ^b^	33.1 ± 4.47 ^a^	39.8 ± 7.59 ^ab^	39.0 ± 2.27 ^a^	41.7 ± 2.67 ^ab^	42.9 ± 3.33 ^b^
monounsaturation	16.9 ± 5.07 ^b^	9.87 ± 1.92 ^a^	13.9 ± 5.4 ^ab^	19.3 ± 2.59 ^a^	21.0 ± 2.09 ^ab^	23.5 ± 1.43 ^b^
polyunsaturation	23.6 ± 1.45	23.3 ± 5.48	25.9 ± 2.87	19.7 ± 0.85	20.7 ± 1.93	19.3 ± 3.41
n-3	1.67 ± 0.28 ^b^	1.00 ± 0.22 ^a^	0.96 ± 0.35 ^a^	0.79 ± 0.18	0.78 ± 0.14	0.64 ± 0.09
n-6	21.9 ± 1.69	22.3 ± 5.27	24.9 ± 2.60	18.9 ± 0.75	19.9 ± 1.93	18.7 ± 3.35
n-6:n-3	13.5 ± 2.97 ^a^	22.2 ± 1.41 ^b^	28.6 ± 10.14 ^b^	25.1 ± 5.61	26.1 ± 5.03	29.3 ± 4.28
unsaturation index	109 ± 3.85	99.1 ± 20.4	114 ± 15.3	75.6 ± 4.33	78.7 ± 6.48	73.7 ± 7.18
average chain length	18.5 ± 0.51	18.3 ± 0.18	18.6 ± 0.33	17.9 ± 0.11	17.9 ± 0.12	17.9 ± 0.15

n-3, omega-3; n-6, omega-6; n-6:n-3, ratio of omega-6 to omega-3, fatty acid; -, fatty acid below the detection limit or not possible to calculate due to a fatty acid limitation in samples; ^a, b^ values with different letters refer to a significant difference among the treatments (*p* < 0.05).

**Table 8:** The antioxidant enzymes and lipid peroxidation end product of liver and lungs of the experimental piglets (n = 6 animals/treatment). The results represent mean ± standard deviation (SD).

Parameter	Control	15 mg FBs	30 mg FBs	Control	15 mg FBs	30 mg FBs
	Liver			Lung		
GSH (micromol/g prot.)	7.71 ± 0.64	7.49 ± 1.14	7.67 ± 1.19	4.94 ± 0.93	4.76 ± 0.51	4.92 ± 0.93
GPx (U/g prot.)	4.15 ± 0.36	3.88 ± 0.60	4.00 ± 0.54	5.37 ± 0.51	5.53 ± 0.45	5.84 ± 0.53
MDA (nmol/g)	71.0 ± 7.82	65.9 ± 12.0	59.6 ± 9.45	41.6 ± 7.65	43.0 ± 7.09	36.6 ± 2.74

GSH, reduced glutathione; GPx, glutathione peroxidase; MDA, malondialdehyde.

**Table 9:** The proximate and chemical composition of the experimental diet.

Component	Concentration
Crude protein (%)	17.50
Crude fat (%)	3.30
Crude fiber (%)	3.70
Crude ash (%)	5.00
Starch (%)	41.8
Lysine (g/kg)	1.11
Methionine (g/kg)	0.37
Ca (g/kg)	0.65
P (g/kg)	0.50
Na (g/kg)	0.18
DE (MJ/kg)	14.7
ME (MJ/kg)	14.1
Fatty acid (methyl ester)	%
C12:0	0.01
C14:0	0.19
C14:1	0.01
C15:0	0.02
C16:0	15.3
C16:1n7	0.33
C17:0	0.08
C18:0	5.61
C18:1n9	26.5
C18:1n7	1.13
C18:2n6	47.1
C18:3n3	2.60
C20:0	0.36
C20:1n9	0.39
C20:2n6	0.07
C20:4n6	0.08
C22:0	0.06
C24:0	0.06
C22:6n3	0.13

## Data Availability

Data is contained within the article.
